# Growing plants on oily, nutrient-poor soil using a native symbiotic fungus

**DOI:** 10.1371/journal.pone.0186704

**Published:** 2017-10-19

**Authors:** Timothy S. Repas, D. Michael Gillis, Zakia Boubakir, Xiaohui Bao, Gary J. Samuels, Susan G. W. Kaminskyj

**Affiliations:** 1 Biology Dept, University of Saskatchewan, Science Place, Saskatoon, SK, Canada; 2 Roy Northern Environmental, Fort St John, BC, Canada; 3 USDA-ARS, Systematic Mycology and Microbiology Lab, Beltsville, MD, United States of America; Universite de Sherbrooke, CANADA

## Abstract

The roots of land plants associate with microbes, including fungal symbionts that can confer abiotic stress tolerance. Bitumen extraction following oil-sand surface mining in the Athabasca region of Alberta, Canada removes plant nutrients but leaves a petrochemical residue, making the coarse tailings (CT) hostile to both plants and microbes. We isolated an endophyte strain of the Ascomycete *Trichoderma harzianum* we call TSTh20-1 (hereafter, TSTh) from a dandelion that was naturally growing on CT. TSTh colonization allowed tomato, wheat, and remediation seed mixtures to germinate and their seedlings to flourish on CT without the use of fertilizer. Compared to control plants, TSTh increased germination speed, percent germination, and biomass accumulation. TSTh also improved plant water use efficiency and drought recovery. TSTh-colonized plants secreted twice the level of peroxidase into CT as did plants alone. Over two months, plants colonized with TSTh doubled the petrochemical mobilization from CT over plants alone, suggesting a peroxide-mediated mechanism for petrochemical degradation. TSTh grew on autoclaved CT, bitumen, and other petrochemicals as sole carbon sources. Further, TSTh is a micro-aerobe that could metabolize ^13^C-phenanthrene to ^13^CO_2_ in 0.5% oxygen. TSTh has excellent potential for contributing to revegetating and remediating petrochemical contamination.

## Introduction

Many soil microbes improve plant productivity and stress resilience, including systemic fungal endophytes that colonize plants without causing disease [1]. Endophytes isolated from plants growing despite abiotic stress can confer habitat-adapted tolerance to that stress when inoculated on seeds or seedlings of otherwise sensitive plants [1–6]. These include bioremediation species, as well as food and animal feed. Abiotic stressors in industrial soils, such as dryness and low-nutrient soils [4], include petrochemicals [7–11].

Lignin and cellulose give plants strength and resilience. Ancient plants were converted by heat and pressure over geological time into petrochemicals [12]. Basidiomycete fungi that metabolize lignin and petrochemicals secrete peroxidases [13–16]. Ascomycetes including *Trichoderma* are known for secreting cellulases [17]. Notably, this genus has repeatedly been isolated from petrochemical contaminated soil [18, 19] and lignin degradation enzymes have recently been identified in *Trichoderma* species [19–21].

Industrial economies have used liquid petrochemicals for more than 150 years. Extraction, refining, transport, storage, end-user sales, and so on, inevitably lead to leaks or spills [12] that create areas of abiotic stress for plants and microbes [7]. Previously, petrochemical contamination was treated by soil removal and storage off site, then replacement with clean soil [8]. This time- and energy-consuming process moved the problem without solving it, and caused environmental disruption. Nowadays, soil bioremediation using local and introduced microbes is preferable, being less disruptive and often less expensive [9–11, 15, 16]. The speed and frequency of spills and leaks exceeds that of cleanup, so this area is ripe for improvement.

The Athabasca oil sands in Alberta, Canada have been surface-mined since the 1960s [8, 22–24]. The important geologic unit is 16-20% bitumen, plus sand, clay and water. Surface-mined bitumen is extracted with hot water [25] plus NaOH or Na-citrate to separate it from byproducts including coarse tailings (CT) [23, 24]. Using either extraction solution, CT had altered pH compared to the original soil (pH 6.6; [3]) (Table 1). The relationship between the high pH of the extraction solutions and the pH of the CT seems counter-intuitive, but relates to solution chemical reactivity. NaOH reacts with the high sulfate in the CT it produces, resulting in a low pH. Na-citrate produces low sulfate CT; the citrate forms buffers that maintain a high pH. Both methods have a bitumen recovery of ~80% [24]. Other byproducts are fine tailings and oil sand process water that present different remediation challenges. In this paper, we will focus on CT revegetation.

**Table 1:** Chemical characteristics of coarse tailing sand, extracted by two methods.

Component / Extraction	Na-citrate	NaOH
Nitrogen (N)	< 4 ppm	< 4 ppm
Phosphorus (P)	< 2 ppm	< 2 ppm
Potassium (K)	< 4 ppm	< 4 ppm
Sulfate	8 ppm	250 ppm
Organic carbon (C)	< 0.6 ppm	not tested
Petrochemicals	516 ppm	481 ppm
Hydrophobic	yes	yes
pH, weathered tailings	8	3.1

CT sites must be reclaimed to self-sustaining boreal forest [8, 22–24]. Reclamation includes revegetation, remediation and, eventually, certification. CT are difficult to revegetate because they lack many plant nutrients, have altered pH compared to boreal forest sites, and residual petrochemicals that make them hydrophobic [Table 1, S1 Fig]. Costs for CT reclamation are seldom disclosed. In his 2010 report, Devenny [23] said that reclamation of disturbed ground (CT) was arbitrarily set at $30,000 per hectare. In contrast at an experimental CT site described at a meeting in 2015, 17 ha (100 hectares = 1 km^2^) had been revegetated for US$50 million (Kaminskyj, *pers comm*), about $3 million per hectare, 100-fold more than the estimate. If typical, this latter cost would be prohibitive over hundreds of km^2^. CT and petrochemical leaks and spills share potential bioremediation solutions, but cost estimates vary widely.

We describe a strain of *Trichoderma harzianum*, TSTh20-1 (TSTh), that was isolated as an endophyte from a dandelion found growing on CT. *Trichoderma harzianum* is a globally distributed soil fungus and a plant endophyte [17]. TSTh-inoculated seeds germinated and seedlings grew well on CT, and particularly when stressed they grew faster than uncolonized plants. In culture, TSTh grew and sporulated on bitumen, crude oil, diesel oil, and other substrates as sole carbon sources. Also, TSTh grew on ^13^C-phenanthrene as a sole carbon source under micro-aerobic conditions, producing ^13^CO_2_. As a plant endophyte and saprotroph, TSTh has great promise for contributing to revegetation and reclamation of CT and soil after petrochemical leaks/spills.

## Results and discussion

This is the first report that a strain of the Ascomycete *Trichoderma harzianum*, TSTh20-1 (TSTh), is a systemic endophyte that promotes plant growth on nutrient-poor, petrochemical-contaminated soil.

### Chemistry of coarse tailings (CT)

Chemical analysis of CT following NaOH or Na-citrate extraction reflected conditions where our TSTh-source dandelion was collected (Table 1). Neither type of CT had detectable NPK, each had ~500 ppm petrochemical residue, and both were hydrophobic. As discussed above, CT had altered pH compared to the original soil (pH 6.6) [3] with either extraction solution (Table 1). CT are barren when first deposited, then after a decade or so of weathering CT support growth of weedy pioneer plants. We used barren CT from both extraction methods for experiments in this study, and found that TSTh performed equally well.

The mineral soil in forests overlying oil sands is hydrophilic (Panel A in S1 Fig), since water droplets soaked in before they could be photographed. In contrast, CT are hydrophobic (Panel B in S1 Fig), as is mineral soil from remediated CT (Panel C in S1 Fig). The contact angle for the droplets was about 100° (Panel D in S1 Fig).

Despite supporting a self-sustaining a boreal forest, the mineral soil of remediated CT remained hydrophobic. Although clearly no longer a problem after remediation, it is highly likely that the hydrophobicity of barren CT would hamper early-stage revegetation. We will present evidence that TSTh–inoculated seeds and seedlings can germinate and grow directly on CT, as well as contributing to petrochemical degradation.

### Isolation and identification of a *Trichoderma harzianum* strain

**Fig 1:**
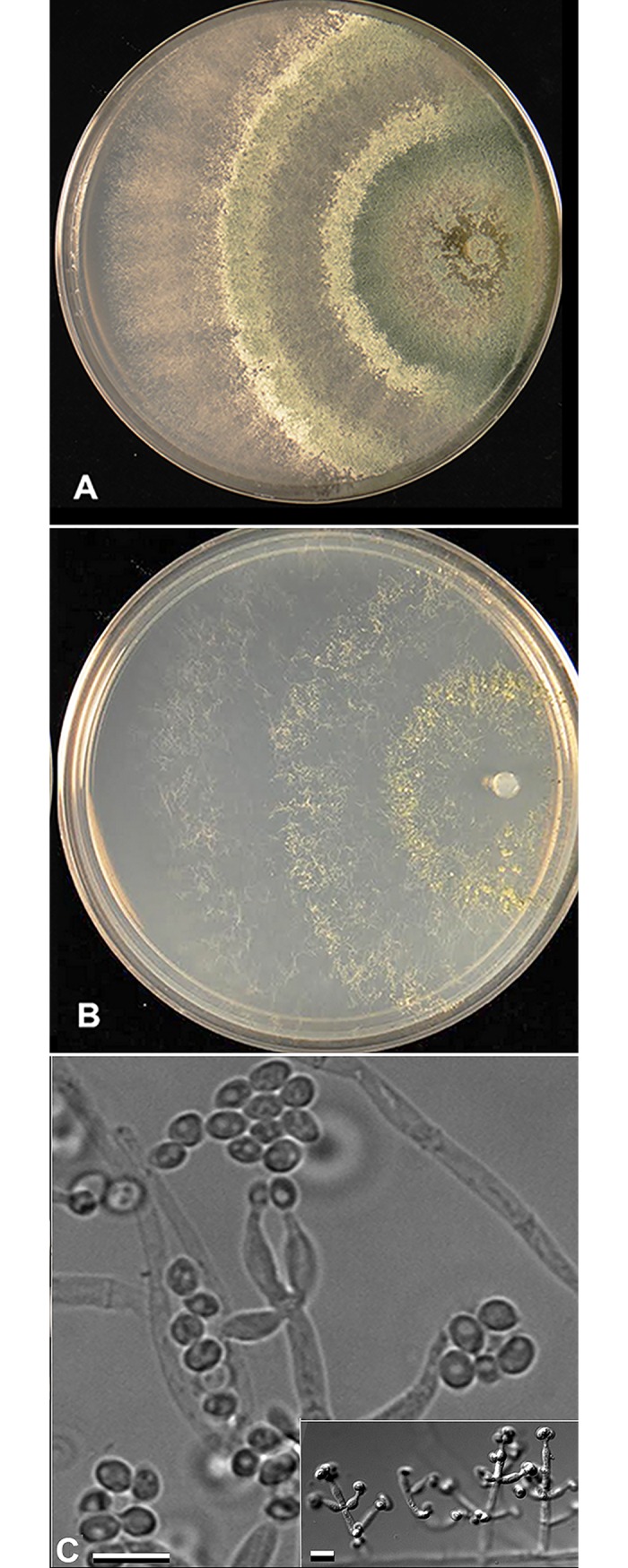
*Trichoderma harzianum* TSTH20-1 (TSTh) culture and conidiation morphologies. TSTh20 growing on A) 100% potato dextrose agar, B) synthetic nutrient-poor agar (SNA). C) TSTh conidiophore on 10% PDA. Bar represents 5 μm. Inset: TSTh conidiophores on 100% PDA. Bar represents 20 μm.

In 2007, endophyte fungi were isolated from a dandelion (*Taraxacum officinale* L.) that had been growing in isolation on barren CT [3]. Of four endophytes isolated, only the *Trichoderma* strain was able to confer tolerance for plant growth on CT. *Trichoderma* identification was assessed by colony appearance on 100% PDA (Fig 1A) and SNA (Fig 1B), by conidiation morphology (Fig 1C), and by ITS sequencing Bao [3] For precise identification, partial sequences of the diagnostic protein translation-elongation factor 1-alpha (*tef1*) were sequenced for two cultures of TSTh. BLAST (https://blast.ncbi.nlm.nih.gov/Blast.cgi) revealed them to be 99% identical to the ex-type and other cultures of *T*. *harzianum* [26]. Sequences were deposited as GJS 08–200 (GenBank KY677912) and GJS 08-201 (GenBank KY677911). *Trichoderma harzianum* TSTh20-1 is stored at the American Type Culture Collection (www.atcc.org) as PTA-10317, US patent 8,598,083.

### TSTh increased seed germination and seedling growth

**Fig 2:**
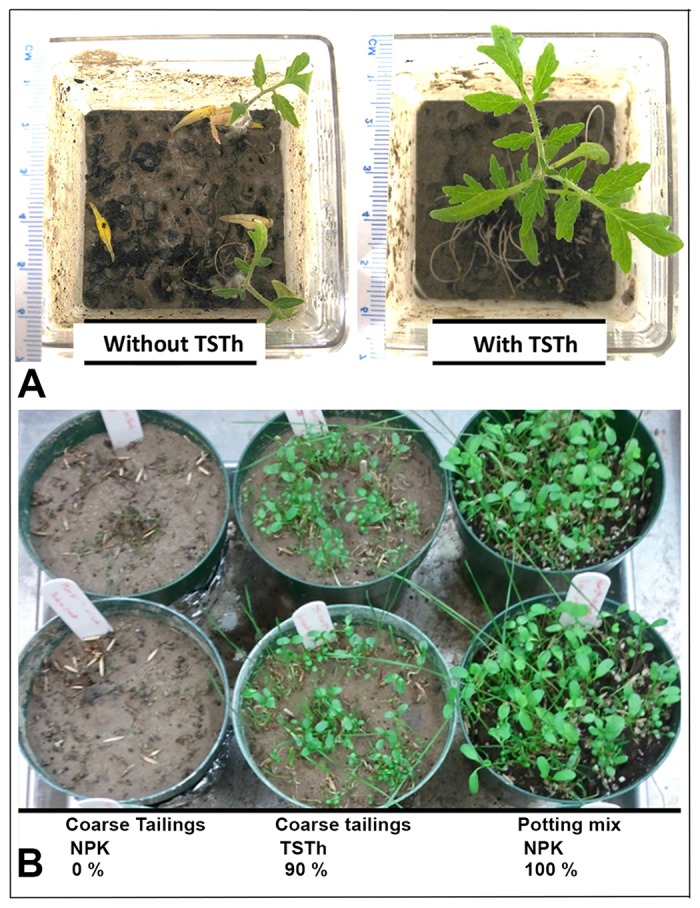
Plant growth is improved with *Trichoderma harzianum* TSTh20-1. A) Two week old control (left) or TSTh-inoculated (right) tomato seedlings were transplanted to coarse tailings (CT), then given ultrapure water for an additional 2 weeks. B) Equal numbers of white clover and slender wheat grass seeds were scattered on CT (left, middle) or potting mix (right). Left- and right-hand pots were treated with NPK. TSTh-treated seeds on CT (middle) were given ultrapure water. Pots were photographed at 3 weeks.

Two-week-old tomato seedlings grown on potting mix were treated with sterile water or TSTh spore suspension, then transplanted to autoclaved CT for two weeks (3). Mock-inoculated seedlings barely survived (Fig 2A, left), whereas tomatoes colonized with TSTh thrived even when watered with ultrapure water (Fig 2A, right). Tomato seedlings from 2 sets of 5 replicate Magenta boxes were washed free of CT. The average fresh weight of a 4 week-old TSTh-colonized tomato plant (560 ± 50 mg) was significantly higher than a control seedling (200 ± 60 mg)(*t*-test, P<0.0001). The dry weight per seedling was also significantly higher for the TSTh-colonized seedlings (260 ± 3 mg) than for axenic plants (140 ± 2 mg)(*t*-test, P = 0.001). TSTh increased the fresh and dry weight of colonized tomato seedlings, suggesting it might also do so for other plant species.

Slender ryegrass and white clover (a standard remediation mix) were seeded (Fig 2B) onto CT or potting mix. All of the seeds germinated on potting mix watered with 1% NPK 20:20:20 (Fig 2B right), as expected. However, none of the seeds germinated on CT, even with fertilizer (Fig 2B left). In contrast, seeds on CT inoculated with TSTh showed > 90% seed germination and much better growth than the control pots, even though they were watered with ultrapure water (Fig 2B centre). We found similar results when using a commercial ‘BC Forest Bioremediation Mix’ of six monocot and legume seeds.

Wheat germination was assessed in damp sand plus sterile or TSTh-treated charcoal. Seedlings were sampled from 2–5 d after seeding, by washing the roots free of sand and then imaging. The longest root from each seedling was measured to the nearest mm. TSTh-colonized plants had significantly longer roots than control plants (P = 0.0206, *t-*test).

These results show first that TSTh allowed seeds to germinate on directly CT watered with ultrapure water, whereas those on axenic CT watered with 1% NPK did not germinate. Current revegetation methods typically cover the CT with a layer of peat that is seeded and fertilized, sometimes more than once. With TSTh, CT can be seeded directly. Second, early nutrient allocation in TSTh-treated wheat seedlings favoured roots over shoots, which has also been shown for rice [4]. Rapid root system growth is important for seedling establishment, especially in semi-arid conditions like the Canadian Prairies. Third, TSTh-inoculated tomatoes grew significantly faster on CT than axenic seedlings. All of these factors affect seedling establishment, particularly where there are several abiotic stressors. We interpret this to strongly suggest that TSTh could contribute to CT revegetation and to bioremediation.

### TSTh increased water-use efficiency and drought recovery

**Fig 3:**
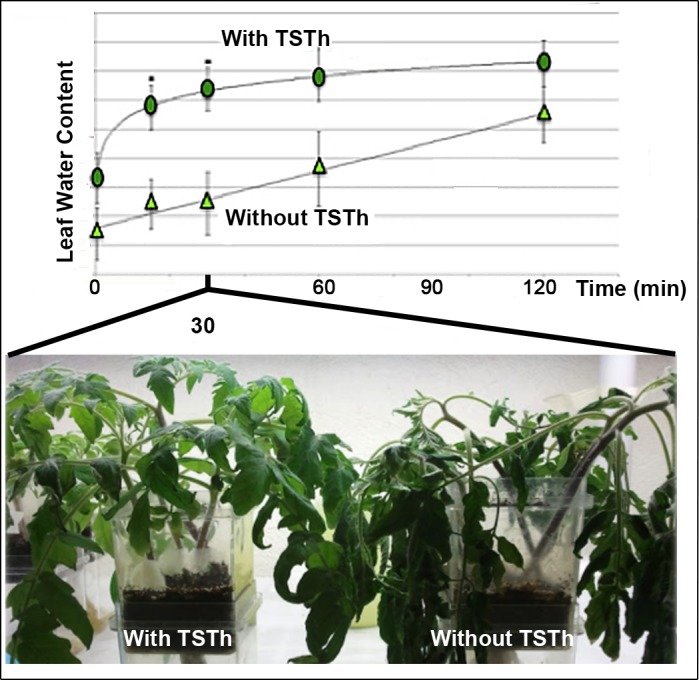
TSTh improved drought recovery for tomato seedlings. Six-week-old tomato seedlings were deprived of water for 2 d to induce wilt, then watered heavily. The graph shows leaf water content prior to and during recovery. Plants colonized with TSTh (ovals) wilted less and recovered faster than those without (triangles). The image shows recovery at 30 min. ‘With TSTh’ regression: y = 0.165 ln(x) + 88.9, r^2^ = 0.9988. ‘Without TSTh’ regression: y = 0.016 x + 87.79, r^2^ = 0.981.

Tomatoes were grown for six weeks in potting mix with or without TSTh. Plants were deprived of water (the bottom chambers of the Magenta boxes were emptied) for 2 d to induce wilt. Leaf disks were taken from representative leaves of both treatment groups for water content analysis at the end of the drought treatment and during recovery after thorough watering. The TSTh-treated plants wilted less during the drought, and recovered much faster (at a logarithmic rather than arithmetic rate) after watering (Fig 3). Notably, the TSTh-treated leaves had similar water content *before* the recovery period as the axenic plants had *after* 60 min of watering. Plants inoculated with TSTh had better wilt resistance and drought recovery, which is important where rain is sporadic and (or) the substrate is hydrophobic.

### TSTh increased peroxidase secretion

**Fig 4:**
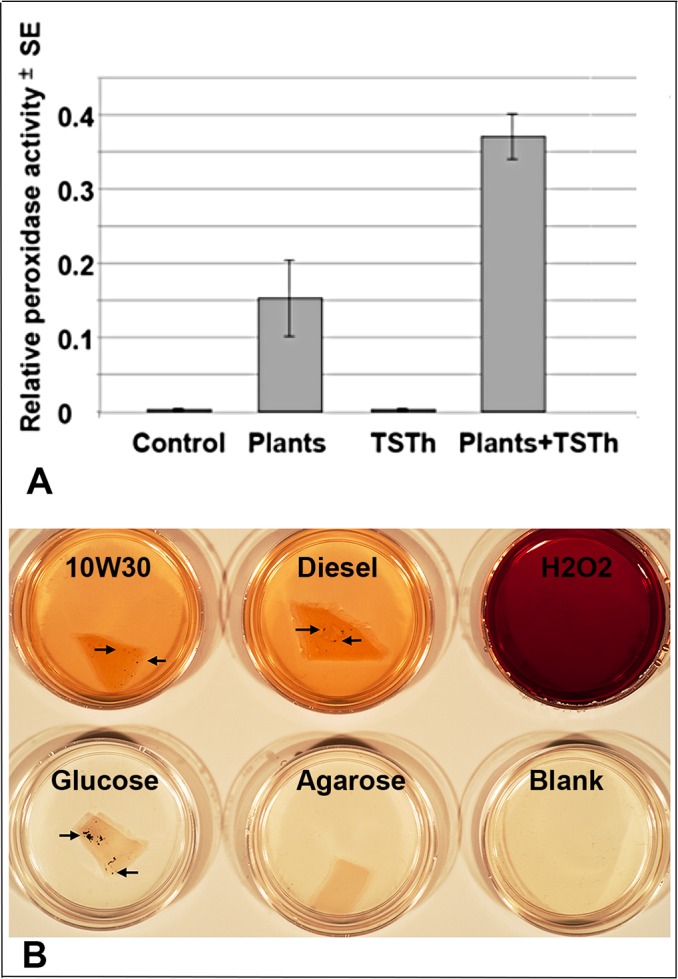
Secretion of reactive oxygen species. A) When grown on coarse tailings, both axenic plants and those colonized with TSTh secreted cell-free peroxidases into the soil. TSTh colonization was associated with a more than 2-fold increase in peroxidase secretion. B) TSTh was assayed with diaminobenzidine. As well as the H_2_O_2_ positive control, TSTh grown on 10W30 motor oil and on diesel oil were positive for ROS, whereas TSTh grown on glucose was not. Cultures were grown until sporulation (arrows).

We grew remediation plants on CT with or without TSTh for 2 months, then assayed the CT for peroxidase [27]. Plants did secrete peroxidase, however plants colonized by TSTh secreted more than twice as much as plants alone (Fig 4A). TSTh colonization increased cell-free peroxidase secretion by plant roots.

Due to the relatively low biomass of TSTh compared to plant roots, we used a more sensitive method to assay for reactive oxygen species (ROS) produced by TSTh alone. Diaminobenzidine (DAB) produces a brown compound in the presence of ROS, which can be generated by peroxidases [28]. We compared cultures grown on glucose, 10W30 and diesel oil, each in agarose plus mineral salts (Fig 4B). Controls were uninoculated agarose, a positive H_2_O_2_ control, and a DAB solution blank. The positive control had the darkest colour, followed by TSTh grown on 10W30 oil or diesel oil, and finally the colony grown on glucose. The agarose and the DAB solution blanks had similar pale colour (Fig 4B). This strongly suggests that TSTh does produce ROS given the appropriate nutritional environment [29].

**Fig 5:**
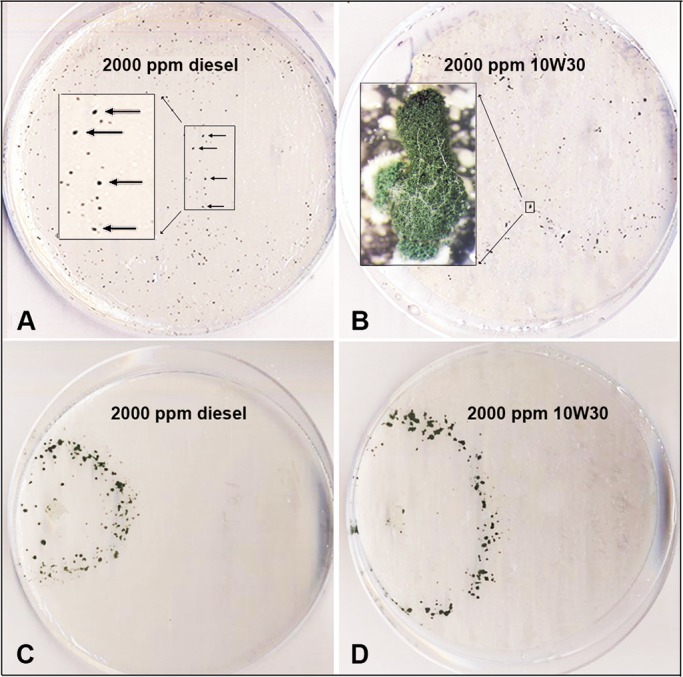
*Trichoderma harzianum* TSTh20-1 (TSTh) growing for 14 d (A, B) or 7 d (C, D) on diesel and 10W30 oil, respectively. TSTh in A, B had been growing for 5 weeks on petrochemicals, whereas C, D had been growing for 16 weeks. Box in A indicates tiny spore aggregates (ca 10^5^ spores) on diesel. Box in B indicates a larger spore aggregate (ca 5x10^6^) on 10W30. Growth and sporulation was much improved in Fig 5C, D.

**Fig 6:**
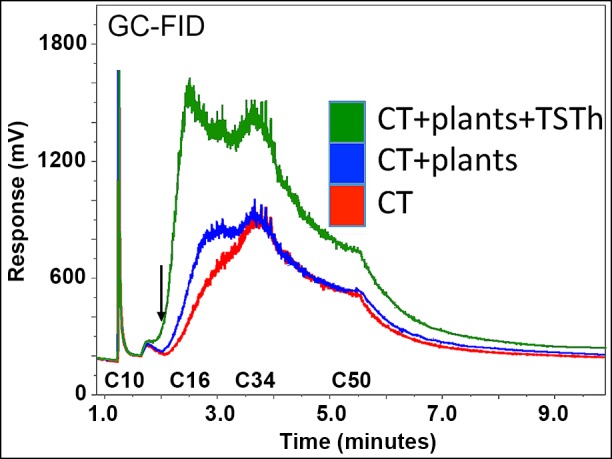
Plants colonized with *Trichoderma harzianum* TSTh20-1 (TSTh) mobilized petrochemicals across a broad size range. Plants colonized with TSTh (area under the green [top] trace) had twice the petrochemical mobilization as plants alone (area under blue [middle] trace), and tailing sand (TS; area under red [bottom] trace). Petrochemicals larger than C14 (arrow) are not volatile.

**Fig 7:**
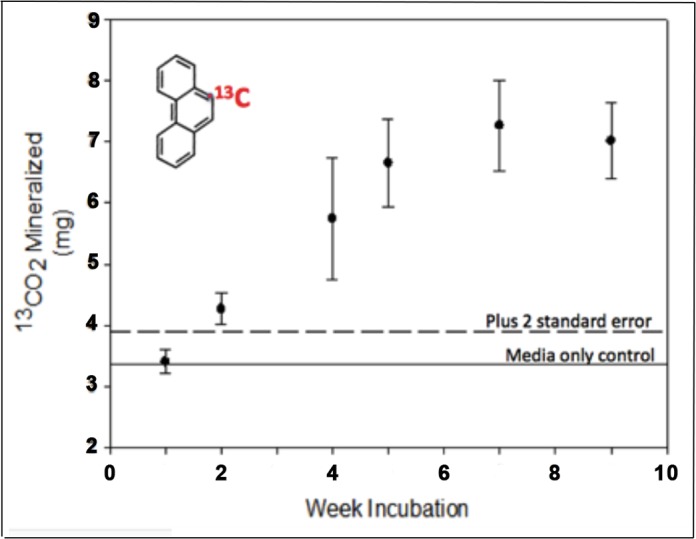
*Trichoderma harzianum* TSTh20-1 (TSTh) metabolized ^13^C-phenanthrene to ^13^CO_2_ under micro-aerobic conditions. After two weeks growth and for more than nine weeks, TSTh generated several-fold more ^13^CO_2_ than the media-only control treatment (solid line) or that plus two standard errors (dashed line).

Petrochemicals and lignins are chemically complex [12]. Peroxidases are ROS-mediating enzymes secreted by white rot fungi [14], which can grow on both substrates. The mechanism(s) by which TSTh modulates plant metabolism to increase peroxidase secretion could include epigenetic regulation [5] Additional possibilities are discussed in Chapter 2 of [17]. TSTh has been shown to have a suite of comparable enzymes [16,18–21] (Figs 5 and 6, S2 Fig) that can degrade these compounds, and generate ROS when grown on petrochemicals. Unlike hydrolases, peroxidases are effective on more than one type of chemical bond, including those in polyaromatic hydrocarbons [7, 9–11] (Fig 7). Fernández-Fueyo et al. [29] showed that level of peroxidase expression in the white rot fungus, *Pleurotus ostreatus* depended on medium composition. We showed that TSTh hyphae grown on diesel or 10W30 motor oil secreted ROS into the medium (the agar and DAB solution were stained), whereas TSTh grown on glucose did not, consistent with [29]. TSTh-colonized plants as well as TSTh itself secreted much more peroxidase in the presence of petrochemicals.

### TSTh grew on petrochemicals as sole carbon sources

*Trichoderma* spp have frequently been isolated from petrochemical contaminated soil [30, 31]. However, attempts to grow fungi on petrochemicals as *sole* carbon sources are relatively few *e*. *g*. [18].

Until our study, research on systemic endophytes had not included an abiotic stressor that could also be a carbon source [2, 4]. We inoculated bitumen (S2 Fig) and CT with TSTh, each of which sporulated in a few days. Samples of sporulation on CT and bitumen were plated on 10% PDA, where they produced colonies characteristic of *Trichoderma*. No colonies grew from uninoculated TS.

TSTh grew and sporulated on bitumen (S2 Fig), 2000 ppm diesel oil (Fig 5A and 5C) and 2000 ppm 10W30 oil (Fig 5B and 5D). TSTh also grew and sporulated on diverse substrates as sole C sources (lubricating grease, roofing tar, Vaseline, butter, sesame oil). Growth and sporulation on low nutrient sources was comparable to Fig 5A and 5B. Over time, both growth and sporulation improved for 10W30 oil and diesel oil. Notably, TSTh isolates that had been barely able to sporulate on diesel and 10W30 oil in May 2017 grew and sporulated much better on the same substrates in July 2017 (cf Fig 5C and 5D with Fig 5A and 5B). We interpret this as a mutagenic adaptation to long-term growth on petrochemicals.

We acquired soil samples from Fort St John, BC that were contaminated with 5000 ppm crude oil. Triplicate 1 g samples were suspended in 25 mL of sterile mineral medium, in 50 mL conical flasks. These were inoculated with TSTh and incubated at room temperature for 4 weeks with shaking at 150 rpm. Samples of the medium taken before and after the 4-week incubation were analyzed for C10-C19 (down by 90% at 4 weeks) and C19-C32 (down by 75% at 4 weeks). TSTh significantly reduced petrochemical content for small and medium petrochemical fractions. It is possible that the C10-C19 content was further reduced by volatilization of molecules smaller than C14 during 4 weeks agitation.

TSTh was able to grow as a saprotroph on diesel oil and 10W30 motor oil. TSTh was also able to greatly reduce small and medium petrochemical fractions in crude oil. Preliminary evidence for germination and growth of wheat on diesel-contaminated potting mix also showed better seedling growth of plants that were colonized by TSTh.

### TSTh-colonization increased plant mobilization of petrochemicals from CT

Because plants colonized with TSTh grew well on CT, we assessed the petrochemical profiles of CT, CT with plants, and CT with plants colonized by TSTh after 2 months growth. The GC-FID assay we used was quantitative for petrochemicals with fewer than 50 carbons (C50), and qualitative for larger molecules [32]. The petrochemical residue in CT likely includes large, recalcitrant petrochemicals [10, 12], some of which are >C250 [12].

Compared to CT alone (Fig 6, area under the red [lowest] trace), CT supporting plant growth (Fig 6, area under the blue [middle] trace) had more C16-C32 content. Notably, plants colonized with TSTh (Fig 6, green [upper] trace) had twice the C14-C32 content as CT with plants alone. This suggests there was a pool of unknown extent of un-assayable >C50 molecules that were being converted into <C50 fragments, which then could be assayed quantitatively. Taken together, plant root peroxidase secretion, particularly for plants colonized with TSTh, contributed to breaking down the petrochemical residues in CT. TSTh appeared to induce the plants it colonized to mineralize petrochemicals with higher efficiency [17]. As well as quantitative differences between the three sample types in C10-C50 petrochemicals, were qualitative differences detected between these samples’ >C50 molecules. Above C50, the trace for plants colonized with TSTh was higher than for plants on CT or CT alone.

### TSTh grew on ^13^C-phenanthrene in micro-aerobic conditions, producing ^13^CO_2_

Soil-dwelling fungi like TSTh [17, 18, 31] are likely to be able to grow at low oxygen tension, since O_2_ in subsoil is consumed by microbes but not replenished from the atmosphere. We tested this *in vitro* using micro-aerobic culture conditions for TSTh plus a polyaromatic hydrocarbon, ^13^C-phenanthrene as a sole carbon source. As well as O_2_, both the NO_3_ and SO_4_ in the mineral solution are efficient reducing agents [33]. Fig 7 shows that by two weeks after inoculation, the TSTh-containing flasks had already produced more ^13^CO_2_ than media-only controls, which continued to increase until nine weeks, when the experiment was terminated. Eukaryotic anaerobic metabolism is discussed in [33]. This is the first demonstration that a *Trichoderma harzianum* strain could metabolize a polyaromatic hydrocarbon in a micro-aerobic environment.

In summary, oil spills and leaks create local environments [10–16] that poison plants and soil microbes alike. CT from oil sand bitumen extraction have lower levels of petrochemical residue than most crude spill sites, since CT is within the upper range acceptable for agricultural soil [8]. However, CT revegetation is problematic, likely due hydrophobicity plus low nutrient levels. TSTh colonizes remediation species, as well as food and feed plants, thereby conferring tolerance to growth on petrochemical-contaminated soils that tend also to be dry and low-nutrient. Each seed or seedling must be treated, which we currently do by mixing seeds with TSTh-treated charcoal grains before sowing them together. We are exploring additional options. TSTh improves seed germination, seedling root growth, water use efficiency, and drought recovery. On petrochemical containing soils, TSTh doubled root peroxidase secretion associated with enhanced degradation and mineralization of petrochemicals. We are confident that TSTh-based technology could reduce the cost of CT revegetation, and over time create a healthy soil microbiome to support ecological succession. We expect anticipate comparable benefits for remediating oil leaks and spills, and brownfield sites.

## Materials and methods

### Media and growth conditions

Most TSTh cultures were grown on potato dextrose agar (100% PDA). For isolating endophyte fungi, surface-sterilized plant parts were plated on 10% potato dextrose agar (10% PDA), supplemented with 50 μg mL^-1^ each of ampicillin, tetracycline, and streptomycin. Some cultures were grown on 2% agarose containing minerals, plus a sole C source.

For identifying TSTh as *Trichoderma harzianum* using culture characteristics, we compared colony morphologies of a validated *T*. *harzianum* strain and TSTh on PDA and on synthetic nutrient-poor agar (SNA). SNA is 1 g KH_2_PO_4_, 1 g KNO_3_, 0.5 g MgSO_4_.7H_2_O, 0.5 g KCl, 0.2 g glucose, 0.2 g sucrose, and 20 g Difco Bacto agar per litre.

For growing TSTh on diesel (Co-Op Fuels) or 10W30 oil (*Certified* ^TM^), 2000–10,000 ppm petrochemical was suspended in molten 2% agarose containing 1% (v/v) mineral salts. These were 10 g (NH_4_)_2_SO_4_, 10 g NaNO_3_, 10 g KH_2_PO_4_, 2.5 g MgCl_2_, 2.5 g CaCl_2_, 1 mL micronutrients (34) per litre.

### Endophyte isolation

Endophyte fungi were isolated from a dandelion (*Taraxacum officinale* L.) that had been growing on CT in 2007 [3]. The roots were washed clean, surface-sterilized for 15 min in 0.6% (w/v) NaOCl, then rinsed exhaustively in sterile water. Lateral roots were cut with sterile scissors into 1 cm-long pieces and plated on 10% PDA with antibiotics. Fungal colonies emerged in 4-7 d. Pure cultures were grown on 100% PDA.

Following each experiment, plant root and shoot samples were tested for TSTh colonization, as for the original isolation.

### Seed inoculation and plant growth

Surface-sterilized tomato (*Solanum lycopersicum* L., var. Rutgers) seeds were inoculated by suspending them in 10^3^–5 x 10^4^ TSTh conidia/mL for 15-30 min, or they were mock-inoculated with ultrapure water. Tomato seeds were planted in double-decker Magenta boxes (MBs; Sigma). The MB chambers were connected by a wick to control water or nutrient composition [4].

Wheat was inoculated either as described for tomato, or using TSTh-treated charcoal (see below). Wheat was planted in 35 mL sand in 50 mL conical tubes, then covered with 10 mL sand. Tubes had one 3 mm hole drilled in the bottom for watering, and one in the cap to retain dampness until coleoptile height was about 5 cm.

Remediation seed mix (grass and clover) was surface sterilized for 10 min in 0.6% NaOCl, suspended in a tea strainer, then rinsed in RO water. Seeds were spread onto 2% agar, and inoculated with TSTh or untreated charcoal.

CT was given a single drench of 1% Tween 20 before sowing seeds.

### TSTh-treated charcoal

A suspension of 8 mL freshly harvested conidia, and 15 mL dry Fluval aquarium charcoal (www.hagen.com) were combined in a 50 mL conical tube, mixed thoroughly to distribute the spore suspension, then air dried at room temperature. We used 8 x 10^5^ spores for 15 mL of charcoal granules to give about 170 spores/granule. Spores were viable for 6 months at 4°C, and longer at -20°C.

### Leaf water content

Five 6 mm diameter disks were punched from representative leaves (one per plant), weighed, dried 48 h at 55°C, then weighed again to assess water content [6].

### Coarse tailing hydrocarbon content

Samples were analyzed for total petroleum hydrocarbons (TPH; petrochemicals in this paper) at ALS Laboratories, Saskatoon SK, using the Canadian Council of Ministers of the Environment (CCME) reference method for the Canada-Wide Standard for Petroleum Hydrocarbons in Soil–Tier 1 Method [32].

### Peroxidase activity

Soil samples were analyzed following the method in [27]. Briefly, 50 g of soil was suspended in 50 mL of 0.05 M NaK-PO_4_ buffer, pH 6.0, shaken for 5 min, then filtered. For the assay, 300 μL of 0.06% H_2_O_2_ in 0.05 M NaK-PO_4_ (pH 6.0), 50 μL 0.5% *o*-dianisidine in methanol, and 2.7 mL soil extract were mixed in a cuvette. Increasing optical density at 460 nm was monitored to determine the change per minute. Blanks were 2.7 mL buffer, or heat inactivated extract. The positive control was purified peroxidase. One unit of peroxidase decomposes 1 μM of H_2_O_2_ per minute.

For TSTh, ROS were assayed using 3,3-diaminobenzidine (DAB; www.goldbio.com) staining modified from [28]. TSTh colonies were grown on 2000 ppm diesel oil or 2000 ppm 10W30 oil in 2% agarose with mineral salts, or on 1% glucose in 2% agarose with mineral salts. Pieces of sporulating colonies were submerged in 2 mg/mL aqueous DAB and incubated for 4 h at 100 rpm, in the light. The positive control was H_2_O_2_, and the negative controls were 2% agarose, and a DAB blank.

### Micro-aerobic metabolism of ^13^C-phenanthrene

TSTh spores were inoculated into autoclaved (sterile, degassed) mineral broth that contained 70 mM NaNO_3_, 11 mM KH_2_PO_4_, 6 mM KCl, 2 mM MgSO_4_, 29 μM MnCl_2_.4H_2_O, 17 μM H_3_BO_3_, 8 μM ZnSO_4_.7H_2_O, 7 μM CoCl_2_.6H_2_O, 2 μM FeSO_4_.7H_2_O, 1 μM CuSO_4_.5H_2_O, 1 μM Na_2_MoO_4_.2H_2_O [34]. Experimental flasks each contained 33 mg ^13^C-phenanthrene (Sigma) as a sole carbon source. All flasks were purged several times with research grade N_2_ (Praxair, 0.5% O_2_ v/v) before the start of incubation.

The ^13^CO_2_ produced by triplicate samples of media-only control and TSTh-inoculated flasks was measured weekly using a Picarro G2101-I analyzer (www.picarro.com/assets/docs/CRDS_Analyzer_for_Isotopic_CO2_in_Ambient_Air_-_Model_G2101-i.pdf), that had been modified with a sample port to inject gas samples.

### Photography

Cultures and seedlings were imaged using a Epson Perfection P3200 Photo flatbed scanner. The DAB-stained sample images were taken with a Nikon D100 SLR camera. The large TSTh spore aggregate and sporulation on bitumen was imaged using a Wild dissection microscope and a DynoEye ocular camera (www.BigC.com). The TSTh conidiophores were imaged with a Zeiss AxioImager.Z1.

## Supporting information

S1 Fig Hydrophobicity of mineral soils before and after bitumen extraction. A) Native boreal forest soils are hydrophilic: water droplets formed damp spots before they could be photographed. B) Extracted CT were hydrophobic, as were C) remediated CT. D) How contact angle is measured.
(PDF)

S2 Fig *Trichoderma harzianum* TSTh20-1 sporulating (arrows) on bitumen after 5 d. The bitumen and the charcoal-TSTh inoculum (top) were pressed onto agarose. Bar represents 2 mm.
(PDF)
