# Supplementary material for: Growing plants on oily, nutrient-poor soil using a native symbiotic fungus
Source: PLoS One. 2017 Oct 19;12(10):e0186704. doi: 10.1371/journal.pone.0186704 (PMC5648232; doi:10.1371/journal.pone.0186704)
Supplement: S1 Fig — A) Native boreal forest soils are hydrophilic: water droplets formed damp spots before they could be photographed. B) Extracted CT were hydrophobic, as were C) remediated CT. D) How contact angle is measured. (PDF) [file pone.0186704.s001.pdf]

Repas et al, Growing plants on oily, nutrient poor soil using a native symbiotic fungus.

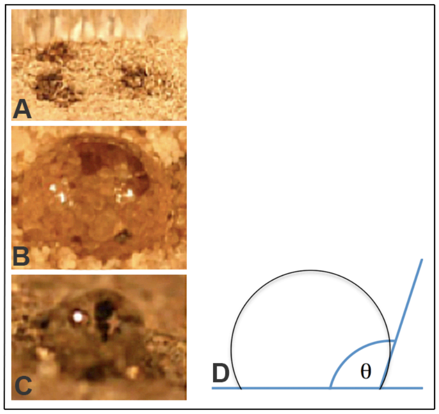

S1 Fig. Hydrophobicity of mineral soils before and after bitumen extraction.

A) Native boreal forest soils are hydrophilic: water droplets formed damp spots before they could be photographed. B) Extracted CT were hydrophobic, as were C) remediated CT. D) How contact angle is measured.
