# Supplementary material for: Growing plants on oily, nutrient-poor soil using a native symbiotic fungus
Source: PLoS One. 2017 Oct 19;12(10):e0186704. doi: 10.1371/journal.pone.0186704 (PMC5648232; doi:10.1371/journal.pone.0186704)
Supplement: S2 Fig — The bitumen and the charcoal-TSTh inoculum (top) were pressed onto agarose. Bar represents 2 mm. (PDF) [file pone.0186704.s002.pdf]

Repas et al, Growing plants on oily, nutrient poor soil using a native symbiotic fungus.

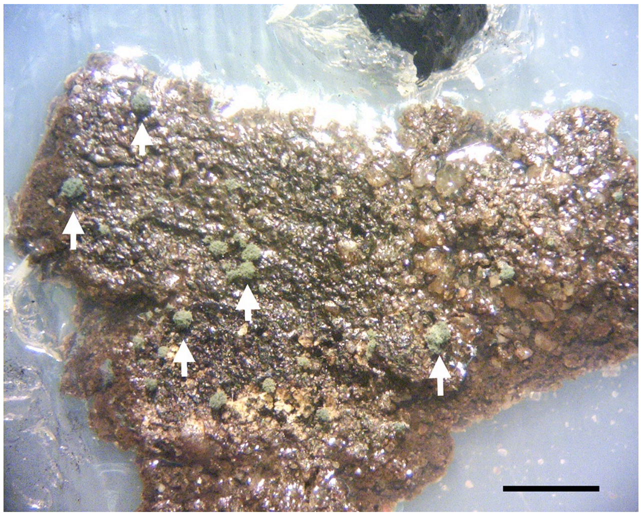

S2 Fig. *Trichoderma harzianum* TSTh20-1 (TSTh) sporulating (arrows) on bitumen after 7 d. The bitumen and the charcoal-TSTh inoculum (top) are pressed onto 2 % agarose. Bar represents 2 mm.
